# Introduction of a spectrophotometric method for salivary iodine determination on microplate based on Sandell-Kolthoff reaction

**DOI:** 10.2478/raon-2024-0035

**Published:** 2024-07-22

**Authors:** Adrijana Oblak, Jernej Imperl, Mitja Kolar, Gregor Marolt, Blaz Krhin, Katja Zaletel, Simona Gaberscek

**Affiliations:** Division of Nuclear Medicine, University Medical Centre Ljubljana, Ljubljana, Slovenia; Faculty of Medicine, University of Ljubljana, Ljubljana, Slovenia; Faculty of Chemistry and Chemical Technology, University of Ljubljana, Ljubljana, Slovenia

**Keywords:** iodine, salivary iodine concentration, Sandell-Kolthoff reaction, Inductively Coupled Plasma Mass Spectrometry

## Abstract

**Background:**

Iodine is an essential element for the synthesis of thyroid hormones. Therefore, a reliable marker of iodine supply is important. Iodine is predominantly excreted via kidneys, but also via salivary glands. Our aim was to introduce a new and simple method for determination of salivary iodine concentration (SLIC).

**Materials and methods:**

Self-prepared chemicals and standards for Sandell-Kolthoff reaction on microplate with ammonium peroxydisulfate (AP) in the range 0−400 µg/L were used. Suitability of water-based standards (WBS) and artificial saliva-based standards (ASS) for standard curve were tested. We followed standards for method validation, defined concentration of used AP and compared our results with Inductively Coupled Plasma Mass Spectrometry (ICP-MS).

**Results:**

WBS gave more reliable results than ASS as an underestimation of iodine concentration was found for ASS. LoB was 6.5 µg/L, LoD 12.0 µg/L, therefore analytical range was 12−400 µg/L. Intra- and inter-assay imprecisions at iodine concentrations, namely 20, 100, 165, and 350 µg/L were 18.4, 5.1, 5.7, and 2.8%, respectively, and 20.7, 6.7, 5.1, and 4.3%, respectively. Suitable molarity of AP was 1.0 mol/L and showed no difference to 1.5 mol/L (P values for samples with concentration 40, 100, and 150 µg/L, were 0.761, 0.085, and 0.275, respectively), whereas there was a significant change using 0.5 mol/L (P<0.001). Saliva samples could be diluted up to 1:8. There was no interference of thiocyanate and caffeine up to 193.5 mg/L. Our original method was comparable to ICP-MS. Spaerman coefficient was 0.989 (95% CI: 0.984−0.993).

**Conclusions:**

The new method for SLIC determination is in excellent agreement with ICP-MS and easy-to-use.

## Introduction

Iodine is an essential trace element for the synthesis of thyroid hormones thyroxine and triiodothyronine, which importantly influence human development and metabolism. The human body contains up to approximately 20 mg of iodine, of which approximately 15 mg is stored in the thyroid gland.^1^ Iodine also accumulates in choroid plexus, gastric mucosa, mammary glands during lactation, and in salivary glands.^1,2^ With adequate iodine supply, more than 90% of iodine is excreted via urine.^3^ Small amounts of iodine are excreted via feces, sweat, and saliva. With iodine deficiency, less iodine is excreted and more is accumulated in the thyroid gland.^4^ Iodine supply significantly affects the incidence and severity of thyroid diseases.^5^ Therefore, reliable biomarkers are needed for the assessment of iodine supply. Determination of urinary iodine concentration (UIC) is most widely used. But it has one major drawback: it requires collecting a 24-hour urine sample, as a single spot urine sample is subject to diurnal variations in UIC and is therefore not directly comparable to 24-hour urinary iodine excretion.^6^ In salivary glands, iodine is transported from blood to saliva via the Na^+^/I^−^ symporter.^7,8^ Thus, salivary iodine could be a biomarker of iodine supply. Saliva samples are easier to obtain and transport than urine samples.

Iodine concentration can be determined using different methods, where Inductively Coupled Plasma with Mass Spectrometry (ICP-MS) is considered a gold standard method. More accessible are spectrophotometric methods with different approaches to remove potential interferences and organic matter in the samples, using different digestion methods followed by manual or automatic Sandell-Kolthoff (S-K) reaction, where catalytic effect of iodide is used to quantify iodine in the sample. Digestion method using chloric(VII) acid has its drawbacks, because it represents a potential hazard, analyses should be performed in fume hoods and HI or I_2_ are formed, thereby falsely leading to underestimation of final results. On the other hand, ammonium peroxydisulfate is a non-hazardous, non-explosive oxidizing reagent, that has very good analytical performances, as shown by Pino *et al*.^9^

To our knowledge, salivary iodine concentration (SLIC) was measured for the first time using S-K reaction in 2012 by Gulaboglu *et al*., but the samples were digested with chloric acid.^10^ Later, SLIC was only measured by ICP-MS.^11^

ICP-MS is considered the best method for iodine concentration determination, but due to high cost of the instrumentation is not accessible to all laboratories. Our aim was to introduce a simple and low-cost method for determination of SLIC using ammonium peroxydisulfate as an oxidizing reagent followed by S-K reaction on microplate. We have already successfully validated this method for UIC determination.^12^ Therefore, we wanted to use this method on a different sample as well, saliva.

## Materials and methods

### Subjects

Cross-sectional study was conducted at the Division of Nuclear Medicine, University Medical Centre Ljubljana, Slovenia between May 2022 and February 2023. Adult volunteers were invited from local population to participate in the study. Only volunteers without known thyroid diseases were included in the study.

The study was granted by the Republic of Slovenia National Medical Ethics Committee, number 0120-271/2021/3 (valid from 12th of July 2021). Informed consent was obtained from all individuals included in this study.

In all participants, salivary iodine concentration was measured. Saliva was collected into Salivettes® (Sarstedt, Germany) before and 30, 60, and 120 min after any meal during the day. Samples were then centrifuged for 10 min at 1800G at room temperature and frozen at −80°C prior to analysis.

### Materials

Chemicals: potassium iodate(V) (KIO_3_) (Sigma-Aldrich, Germany), ammonium peroxydisulfate ((NH_4_)_2_S_2_O_8_) (Sigma-Aldrich, Germany), deionized water (dH_2_O) (BRAUN, Aqua B. Braun, Sterile, Ecotainer®, Germany), sodium hydroxide (NaOH) (Merck, Germany), arsenic(III) oxide (As_2_O_3_) (Sigma-Aldrich, Germany), sodium chloride (NaCl) (Sigma-Aldrich, Germany), concentrated sulfuric(VI) acid (H_2_SO_4_) (Sigma-Aldrich, Germany), ammonium cerium(IV) sulfate dihydrate ((NH_4_)_4_Ce(SO_4_)_4_×2H_2_O) (Sigma-Aldrich, Germany), 65% nitric(V) acid (HNO_3_) (Honeywell, USA).

All laboratory equipment was treated with 65% nitric(V) acid before preparation of reagents to remove any additional iodine from environment. Preparation of reagents as well as digestion processes and analyses took place in a ventilating fume hood. Reagents were prepared in volumetric flasks. Brief summary of preparation: 1 mol/L ammonium peroxydisulfate solution: dissolution of 228.2 g of (NH_4_)_2_S_2_O_8_ in 1 L of dH_2_O, 0.875 mol/L sodium hydroxide solution: dissolution of 8.75 g of NaOH in 0.25 L of dH_2_O, 0.05 mol/L arsorous acid (H_3_AsO_3_) solution: dissolution of 5 g of As_2_O_3_ in 0.1 L of 0.875 mol/L NaOH on magnetic stirrer. Afterwards, the solution was put in an ice-cold bath and 16 mL of concentrated H_2_SO_4_ was added while stirring on magnetic stirrer. After cooling, 12.5 g of NaCl was added and diluted with dH_2_O up to 0.5 L. Solution was then mixed for 90 min at 60°C. Afterwards the solution was filtrated, 1.75 mol/L sulfuric acid solution: on an ice bath 97 mL of concentrated H_2_SO_4_ was slowly added to 0.5 L of dH_2_O and filled with dH_2_O up to 1 L, 0.019 mol/L ammonium cerium(IV) sulfate solution: dissolution of 6 g of (NH_4_)_4_Ce(SO_4_)_4_×2H_2_O in 0.5 L of 1.75 mol/L H_2_SO_4_.

All reagents were stored in amber bottles in the dark at room temperature except for ammonium peroxydisulfate solution, which was stored in the dark at 2–8°C.

We followed all Clinical Laboratory Standard Institute (CLSI) protocols for method establishment, namely EP06, EP07, EP09, EP15-A3, EP17-A2.

### Preparation of standards

1.68 g of KIO_3_ was dissolved in 1 L of dH_2_O. The solution was then used for preparation of intermediate standard with iodine concentration of 1000 µg/L, which was used later for preparation of standards and control samples (CSs).

Standards were prepared with two different matrices, i.e. as artificial saliva to retain properties comparable to real saliva and as a water solution of iodine, which is used in UIC determination, in order to test appropriateness of matrix for standard curve.

Artificial saliva was prepared according to commercially available products that are available for patients after head and neck cancer treatment for moistening mouth and throat. Ingredients used for the preparation of a 100 mL of artificial saliva were 30.45 mg/mL of sorbitol, 10.15 mg/mL of sodium carboxymethylcellulose, 1.218 mg/mL of potassium chloride, 0.856 mg/mL of sodium chloride, 0.348 mg/mL of potassium hydrogen phosphate, 0.148 mg/mL of calcium chloride dihydrate, 0.052 mg/mL of magnesium chloride hexahydrate, and water for injections as solution with pH set at 7.0. Cellulose was added for adjusting viscosity of matrix retaining properties comparable to real saliva.

Both standards and CSs were prepared and stored according to previously published method for UIC determination.^12^ Briefly, 6 working standards were prepared at concentrations of iodide: 0, 40, 80, 120, 200, and 400 µg/L. For comparison, both standards were measured on six microplates with S-K reaction. Each standard was measured in 6 replicates on each microplate and the mean concentration was calculated, whereas standards in artificial saliva were also analysed by ICP-MS. CSs were prepared at two different levels: 160 and 280 µg/L.

### Preparation of standards

Six pooled saliva samples from different participants were used for testing analytical recovery by adding 10, 20, 40 and 120 µL of intermediate standard with iodine concentration of 1000 µg/L to 500 µL of saliva sample before the digestion step. Samples were measured 8 times and recovery was expressed as the percentage of the measured amount of iodine over the expected amount of iodine.

Three pooled saliva samples from different participants were tested for the potential influence of the molarity of ammonium peroxydisulfate to the final result. For UIC determination, 1 mL of 1.0 mol/L of ammonium peroxydisulfate was used, therefore we tested the same volume of ammonium peroxydisulfate with different concentrations, below and above proposed concentration: 1 mL of 0.5 mol/L, 1.0 mol/L, and 1.5 mol/L.

Linearity of the method was tested using 4 saliva samples, each measured 8 times, in the range up to 165 µg/L. Higher concentrations were not available for linearity testing. Distilled water was used as diluent in ratios of 1:2, 1:4, 1:8, and 1:16. Test was considered as linear, if the recovery was in the range of 80–120%.

The interferences of some potential substances such as thiocyanate and caffeine in the introduced method were tested by adding 19.6, 38.5, 74.1, and 193.5 mg/L of both, potassium thiocyanate and caffeine, to 6 saliva samples. Samples were measured 8 times and results are reported as recoveries expressed as the percentage of the measured over expected amount of iodine. The result was considered as no influences of interferences, if the recovery was in the range of 80–120%.

Limit of blank (LoB) and limit of detection (LoD) of the method were determined. Eighty-eight measurements for LoB from 8 samples on two microplates using two different standard curves were analysed. To test LoB, dH_2_O was used. An F-test was used to compare values of measurements between both microplates. For LoD 180 measurements from 12 samples were analysed on five microplates using five different standard curves. LoD samples were in the concentration range between LoB and 4-times LoB value.

For intra- and inter-assay precision, two different types of samples were prepared. Intra-assay precision was assessed on one microplate where 40 replicates were measured, whereas inter-assay precision was measured on 15 microplates, where at least 5 replicates on each microplate were measured. Reproducibility was assessed by using 4 samples, 3 of which were prepared in water solution at concentration levels 20, 100, and 350 µg/L to cover the range of the assay standard curve, and one was prepared as pooled saliva sample, which was pooled from random saliva samples and final concentration was 165 µg/L. Precisions were measured as coefficients of variation (CV (%)).

### Procedure of the method

All reagents reached room temperature and were gently mixed before use. To 250 µL of standards, CSs, and samples, 1 mL of 1 mol/L ammonium peroxydisulfate solution was added. Oxidation of all samples, including standards and CSs, was performed manually in 13 × 100 mm glass tubes. Solution was then mixed and incubated for 1 h on 95°C on dry bath (ScientificTM IsotempTM Digital Dry Bath/Block Heater, Fisher Scientific, UK). After cooling down to room temperature, 50 µL of standards, CSs, and samples were transferred in duplicate to microplate (Nunc Multiwell plate – 96 well solid flat bottom, Merck, Germany). To each well 100 µL of arsorous acid solution was added. Microplate was sealed and incubated for 60 s on a microplate shaker. Afterwards 50 µL of ammonium cerium(IV) sulfate solution was added to each well within 40 s with an 8-channel semi-automatic pipette. Microplate was sealed and put on the microplate shaker for exactly 30 min. Immediately after shaking, an absorbance measurement at 405 nm on a spectrophotometer (SunriseTM, Tecan, Switzerland) was performed. The results were calculated by plotting the linear optical density (OD) data on Y-axis and linear iodide ion concentration on X-axis. Concentration was inversely proportional to absorbance. Standard curves were developed for each microplate separately. A quadratic polynomial was used to calculate concentration from OD.

### ICP-MS method

For the validation of the introduced method, SLIC was measured at the Faculty of Chemistry and Chemical Technology of the University of Ljubljana by ICP-MS method. The concentrations of iodine were determined by inductively coupled plasma quadrupole mass spectrometer (ICP-MS 7900ce, Agilent Technologies, USA) with the use of internal standards and optimal instrumental parameters, ensuring the lowest detection limits. A forward RF power of 1.5 kW was used with Ar gas flows: carrier 0.85 L/min, makeup 0.28 L/min, plasma 1.0 L/min, cooling 15 L/min, and sample flow rate 0.2 mL/min. Isotope ^127^I was measured in 3 replicates with integration/dwell time of 1 s. Saliva samples were diluted 40-times with ultrapure water (resistivity ≥18.2 MΩ · cm, Synergy Water Purification System, Merck Millipore, Merck, Germany). Working standard solutions were prepared by appropriate dilution of iodide stock standard solution (iodide, 1000 mg/L, CGICI1, Inorganic Ventures, USA) with ultrapure water. Calibration curve was based on 7 working standard solutions in the concentration range 0.1–10 µg/L. Intra-assay and inter-assay imprecisions of saliva samples at concentration levels 40 µg/L were 4.0%, and 5.4%, and at 100 µg/L 5.4%, and 5.7%, respectively.

### Method comparison

ICP-MS method, considered as a reference method, was used for comparison of the methods. One hundred and ten saliva samples were analysed with ICP-MS as well as with the new S-K method.

### Statistical analysis

The Kolmogorov-Smirnov test was used to assess the normality of distribution of analysed data. For comparison of LoB values of measured samples on two different microplates the F-test was used. T-test was used to compare values of different molarity. A P<0.05 was considered statistically significant. Data were expressed as means and standard deviations (SD), as means and ranges when reporting results of recoveries, and for method comparison as medians and ranges. The Passing-Bablok regression analysis and Bland-Altman method were used for the method comparison of analysed data, and the Spearman coefficient was determined. Bias was also assessed. Statistical analysis was done using MedCalc Statistical Software version 20.014 (MedCalc Software Ltd, Belgium).

## Results

**FIGURE 1.**
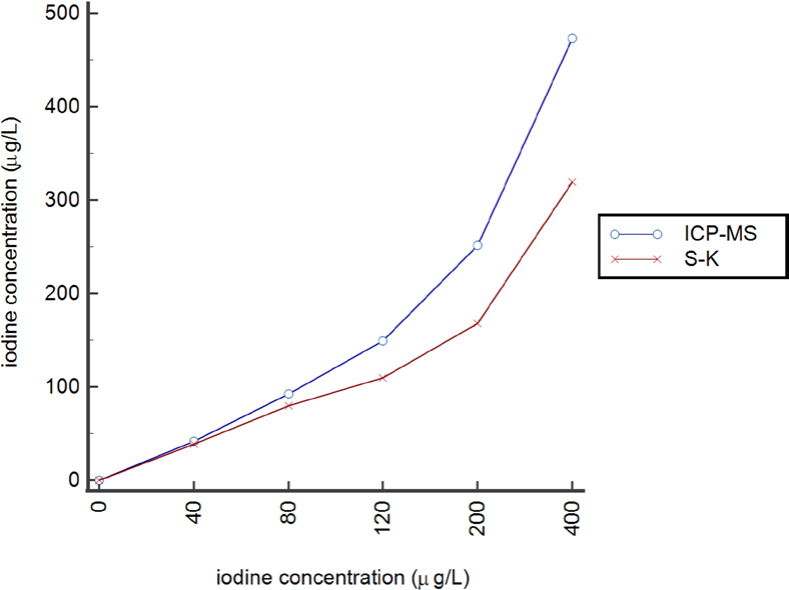
Comparison of measurements of artificial saliva standards (ASS) by two different methods, inductively coupled plasma mass spectrometry (ICP-MS) and Sandell-Kolthoff method (S-K) using ammonium peroxydisulfate on microplate.

To determine the suitability of artificial saliva standards (ASSs) or water-based standards (WBSs) we have measured both types of samples with S-K reaction. Iodine concentrations in ASS were lower than expected in comparison to WBS, as their OD differed up to 26% at highest concentration level. Therefore, ICP-MS was also used for the analysis of ASS. Comparison of ASS measured by S-K method and ICP-MS is presented in Figure 1. We found a significant difference between the methods as, on the average, 37% lower values were obtained with the use of S-K method in comparison to ICP-MS. Therefore, ASSs are not appropriate for iodine determination by S-K method. For further SLIC determination by the new S-K method, only WBSs were used.

Assessment of analytical recovery of 6 different saliva samples spiked with different concentrations of iodine showed no differences between measured and expected iodine concentrations. The mean percentage recovery (range) of iodine at each concentration level of addition of 19.6, 38.5, 74.1, and 193.5 µg/L of stock potassium iodate(V) solution to saliva samples with mean iodine concentration (SD) 34.7 (3.1), 72.2 (2.7), 73.7 (1.5), 113.2 (3.5), 136.0 (3.6), and 146.4 µg/L (3.0), respectively, were 103.0% (95.3–111.4%), 99.5% (94.5–102.6%), 99.5% (97.1–102.8%), 95.2% (90.0–96.9%), 95.4% (91.2–95.7%), and 95.0% (88.6–97.8%), respectively.

**FIGURE 2.**
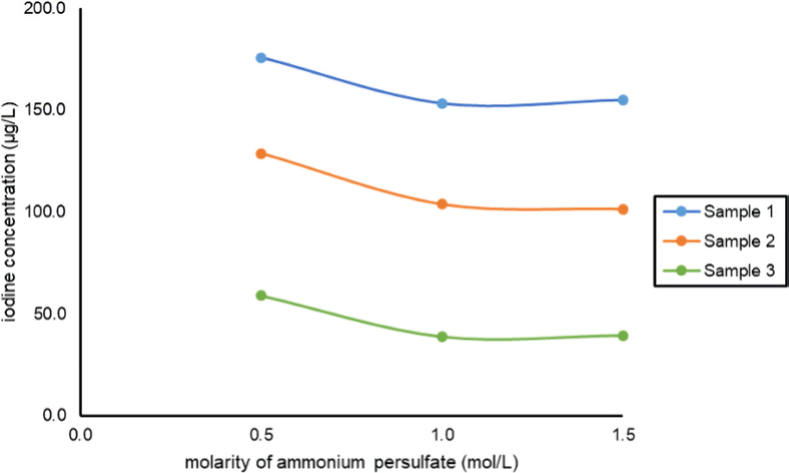
Comparison of different ammonium peroxydisulfate concentrations and their influence on the determined salivary iodine concentration. Comparison between 0.5 and 1.5 mol/L, as well as between 0.5 and 1.0 mol/L for all three samples showed significantly different values (P<0.001) for all three samples, whereas P values for samples 1, 2, and 3, showed no difference between 1.0 and 1.5 mol/L. The corresponding P values were 0.275, 0.085, and 0.761, respectively.

Three saliva samples at concentration levels of 40, 100, and 150 µg/L were tested with 3 different concentrations of ammonium peroxydisulfate, namely 0.5, 1.0, and 1.5 mol/L, respectively. Results are presented in Figure 2. Based on the results, both, 1.0 mol/L and 1.5 mol/L of ammonium peroxydisulfate solution, could be used as oxygenizing agents before the S-K reaction.

LoD for SLIC was 12.0 µg/L, with less than 5% of both false positives (α) and false negatives (β) less than 5%; based on 268 determinations, with 88 blanks and 180 low-level samples; LoB = 6.5 µg/L. LoB data were non-Gaussian. F-test for comparison of different samples for LoB determination showed no statistical difference between the values on different microplates using different standard curves, P = 0.283, so all data for LoB calculation were used. Finally, analytical range was 12–400 µg/L.

The calculated imprecisions expressed as CV (%) for 3 levels of iodine concentrations, at 20, 100, 300 µg/L, and for pooled sample, are presented in Table 1.

**TABLE 1.** Coefficients of variation (CV [%]) for intra- and inter-assay for 4 different iodine concentrations for salivary iodine concentration (SLIC)

**Level**	**Target value, μg/L**	**Number of measurements**	**Intra-assay imprecision, %**	**Number of measurements**	**Inter-assay imprecision, %**
1	20	40	18.4	198	20.7
2	100	40	5.1	192	6.7
3	350	40	2.8	121	4.3
Pooled sample	165	40	5.7	80	5.1

Linearity of the test was assessed on 4 saliva samples. Results are presented in Table 2. The results below the measuring range of the method were omitted from the table.

**TABLE 2.** Dilutions of 4 saliva samples with deionised water. Each sample was measured 8 times and results are presented as mean measured concentration (SD). Recoveries are expressed as the percentages of mean measured amount of iodine over expected amount of iodine after dilution

	**Dilution**	**Measured (M) μg/L (SD)**	**Expected (E) μg/L**	**M/E %**
Sample 1	Non-diluted	105.7 (1.3)	/	/
	1:2	56.9 (1.3)	52.9	107.7
	1:4	28.8 (2.0)	26.4	109
Sample 2	Non-diluted	35.6 (1.0)	/	/
	1:2	19.2 (1.4)	17.8	108.2
Sample 3	Non-diluted	76.7 (1.3)	/	/
	1:2	40.2 (1.7)	38.4	104.8
	1:4	17.3 (1.2)	19.2	90.3
Pooled sample	Non-diluted	165.1 (5.4)	/	/
	1:2	65.0 (3.6)	61.9	105.0
	1:4	33.8 (1.7)	31.0	109.1
	1:8	17.2 (1.6)	15.5	111.1

Recovery tests of 6 different saliva samples spiked with different concentrations of thiocyanate and caffeine showed no significant influence on iodine concentrations. Recoveries are expressed as percentages of observed amount of iodine over expected amount of iodine after potassium thiocyanate or caffeine stock solutions added to samples. The mean percentage recovery (range) of iodine at each concentration level of addition of 19.6, 38.5, 74.1, and 193.5 mg/L of stock solutions of potassium thiocyanate and of caffeine to saliva samples with different iodine concentrations are presented in Table 3.

**TABLE 3.** Recoveries of 6 different saliva samples spiked with 19.6, 38.5, 74.1, and 193.5 mg/L of stock solutions of potassium thiocyanate and of caffeine to saliva samples. Recoveries are expressed as percentages of observed amount of iodine over expected amount of iodine after thiocyanate or caffeine stock solutions added to samples. Each sample was measured 8 times and results are presented as mean salivary iodine concentration (SLIC) (SD) and as the mean percentage recovery (range) of iodine at each concentration level

	**Thiocyanate**		**Caffeine**	

	**SLIC, μg/L (SD)**	**Recovery, % (range)**	**SLIC, μg/L (SD)**	**Recovery, % (range)**
Sample 1	34.7 (3.0)	97.9 (97.3–100.5)	29.1 (1.0)	93.2 (90.7–96.3)
Sample 2	86.0 (7.4)	101.2 (99.0–102.8)	73.0 (1.6)	99.3 (97.0–100.9)
Sample 3	90.7 (3.7)	102.4 (99.0–105.0)	75.2 (1.9)	96.1 (92.0–100.4)
Sample 4	109.0 (3.9)	109.0 (95.9–104.9)	101.3 (3.8)	96.3 (94.1–99.0)
Sample 5	143.0 (2.2)	106.2 (103.8–108.7)	142.1 (2.5)	98.7 (96.1–105.3)
Sample 6	150.6 (3.3)	102.4 (98.2–107.7)	152.8 (3.0)	99.3 (97.3–101.3)

### Comparison of SLIC determination with the S-K method and with the ICP-MS method

**FIGURE 3.**
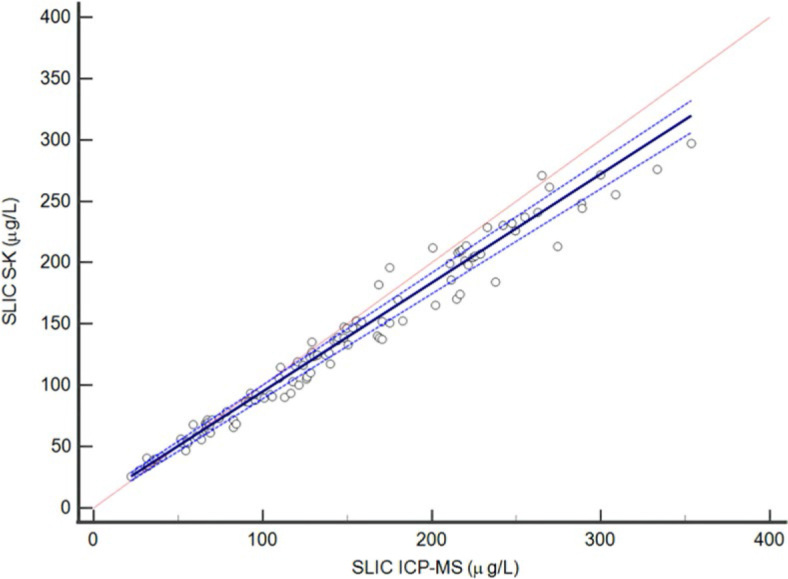
Comparison of salivary iodine concentration (SLIC) determined by the Sandell-Kolthoff (S-K) method using ammonium peroxydisulfate on microplate and by the Inductively Coupled Plasma Mass Spectrometry (ICP-MS) using Passing-Bablock regression. The solid line shows the regression line, dashed lines show 95% confidence interval (CI) for the regression line, and dotted line shows the identity line Y = X. The sample size is N = 110. The regression equation is Y = 0.89*X+6.2. The slope is 0.89 (95% CI, 0.86−0.92), and the intercept is 6.2 (95% CI, 3.3−8.6). The Cusum test for linearity showed no significant deviation from linearity (P = 0.89).

**FIGURE 4.**
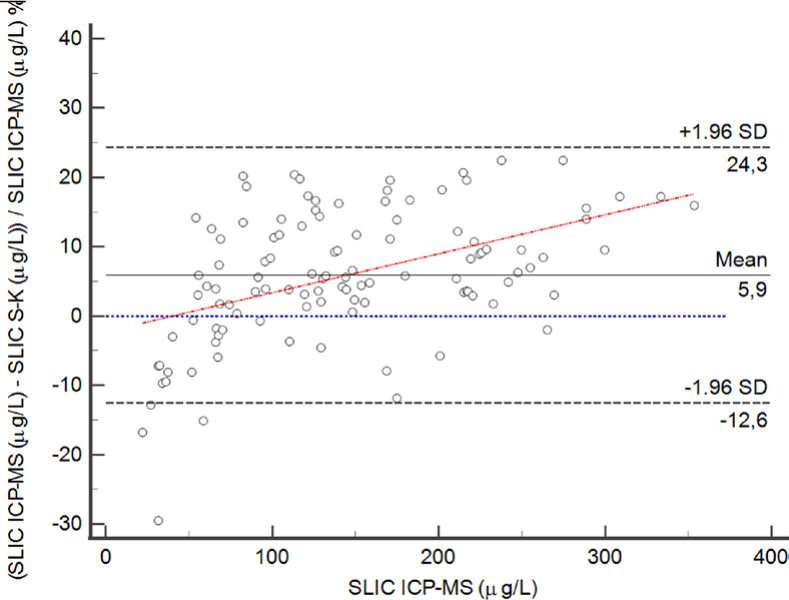
Bland-Altman plot of comparison of salivary iodine concentration (SLIC) determined by the Sandell-Kolthoff method (S-K) and by the Inductively Coupled Plasma Mass Spectrometry (ICP-MS). Differences between the methods are plotted against the ICP-MS method, which is a gold standard method. Sample size is N = 110. Horizontal lines represent the mean SLIC by ICP-MS, zero difference, and 95% confidence interval limits of agreement, which are defined as mean difference plus/minus 1.96 times the standard deviation (SD) of the differences. The mean shows a positive bias of 5.9%.

The 110 samples covered the whole analytical range. For SLIC the median value (range) using the S-K method was 124.1 µg/L (25.8–297.3 µg/L), whilst the median value using the ICP-MS method was 129.8 µg/L (22.1–353.5 µg/L). SLIC results of saliva samples obtained by both methods were not normally distributed, Spaerman coefficient was 0.989 (95% CI: 0.984–0.993), and P<0.001. Passing-Bablok regression analysis and Bland-Altman graph are presented in Figure 3 and Figure 4, respectively. The average bias between the methods was 5.9%. We did not observe any bias at concentrations up to 100 µg/L. With higher iodine concentration, the bias between the methods was 8.2%.

## Discussion

Determination of UIC is a standard method for iodine supply estimation on the population level. Urine sampling, however, is associated with many problems. Because iodine is also excreted in saliva, which is relatively easy to obtain, we developed a simple, easily implementable and low-cost method for determination of SLIC. Our method is in agreement with the reference ICP-MS method. The method is also suitable for measuring SLIC in saliva samples and UIC in urine samples on the same microplate using the same standard curve as it was already shown in our previous paper.^12^

There is very little published data on the determination of SLIC and none of which is based on the method we describe in this work. The method is based on S-K reaction on microplate using ammonium peroxydisulfate as an oxidizing and digestion reagent, which shows sustainability and good performance characteristics.^12^ The main disadvantages are the ‘in-house’ reagent preparation, potential poisonous arsorous acid, and management of arsenic waste. The main advantages of the method are cost-effectiveness, simplicity, and availability of laboratory equipment.

Water based standards were used for UIC determination. To test the saliva matrix effect, standards using artificial saliva were prepared in the same concentration range as WBS. Using ASSs, iodine concentration was underestimated as compared to using WBSs. The results were also confirmed by ICP-MS. We assume that iodine in ASSs binds to organic molecules in artificial saliva and since only available iodide ions participate in S-K reaction, ASSs are not suitable for the use. To verify whether WBSs are appropriate, we have spiked 6 different saliva samples with different amounts of iodine in concentration range 34.7–146.4 µg/L. The average recoveries were very good, 95.0–103.0%, which was within the desirable range 80–120%. For further analysis only WBSs were used. The analytical range was 12–400 µg/L and could be used for both, UIC and SLIC determination on the same microplate, respectively. Measurement range is wide enough to cover expected values in saliva, and the introduced method is sensitive. Although the ICP-MS method has lower limits of quantification, our new method provides sufficient information for SLIC. The method also shows good intra- and inter-assay imprecision at different levels: low, medium, and high. Linearity of the test showed that saliva samples could be diluted up to 1:8 in the concentration range up to 165 µg/L. Higher dilutions could not be tested, since no samples were available in higher concentrations.

The first step of iodine determination was oxidation of samples to obtain accurate measurements with the elimination of interfering substances that could be present in saliva. This reagent – ammonium peroxydisulfate – was already presented by Pino *et al*.^9^ For UIC measurements, 1.0 mol/L of ammonium peroxydisulfate was used. To confirm the suitability of the proposed concentration, we have also tested lower and higher molarities. With 0.5 mol/L of ammonium peroxydisulfate significantly higher iodine concentrations were measured than with 1.0 or 1.5 mol/L (P<0.001 for both). Therefore, 0.5 mol/L proved to be inappropriate. However, no differences were found using either 1.0 or 1.5 mol/L of ammonium peroxydisulfate. In order to be complementary to UIC determination, we continued to use 1.0 mol/L. This way also less ammonium peroxydisulfate is needed, compared to 1.5 mol/L. From the literature it is well known that the disadvantages of the S-K method are the interferences of the substances like iodine, KBr, CuSO_4_, MgSO_4_, nitrites, and ascorbic acid^13,14,15^, which may affect the results. However, the results also depend on the use of oxidizing agent. Ascorbic acid does not affect analysis if ammonium peroxydisulfate is used as the oxidizing reagent, however it affects the result if chloric(VII) acid is used.^9,15^ No method can guarantee the complete removal of all interfering substances. Our focus was on possible interferences of thiocyanate, which is found in cigarettes, and caffeine, which is regularly consumed in the form of caffeinated beverages, and are consequently present in saliva. The thiocyanate influence was already tested^9^, but we used higher concentrations of thiocyanate comparable to those found in saliva of smokers.^16^ The same protocol was followed with caffeine, as higher concentrations than those found in saliva^17^ were tested for possible interference with the assay. Neither thiocyanate nor caffeine had any effect on the assay at concentrations up to 193.5 mg/L. However, possible coloration of saliva with thiocyanate or caffeine could affect the end result since the reaction is based on the absorbance measurement. During the process of digestion (i.e. oxidation), the samples were discoloured, which was observed and verified spectrophotometrically and results were negative (not shown in the Results).

Our method for SLIC determination was compared with a reference ICP-MS method to confirm our results. The comparison showed agreement between the evaluated methods as there was no discrepancy between the two up to 100 µg/L. At higher concentrations we found a slight discrepancy, on average 8.2% underestimation of the introduced method. Saliva samples for ICP-MS analysis were diluted 40 times using MQ to overcome matrix effect. With this in mind, it is plausible to assume that the slight discrepancy between the methods could be due to different media used for SLIC determination.

It is also important to emphasize that there is no External quality assurance assessment scheme for measuring SLIC. Therefore, comparison of SLIC determinations between the laboratories cannot be established yet. We believe the best practice for SLIC determination at this moment would be to verify self-laboratory prepared solutions used for reaction through available external program quality assessment for urinary iodine samples. If measurements are within the range, solutions are acceptable, and are also suitable for the use with saliva samples.

In summary, described spectrophotometric method on microplate using S-K reaction with ammonium peroxydisulfate digestion for SLIC determination shows acceptable performance characteristics and agreement with the reference method ICP-MS, which represents the gold standard. Implemented method showed good sensitivity and is suitable for measuring iodine concentration in various biological samples such as SLIC in saliva samples and UIC in urine samples, where microplate and the standard curve can be used. Method also enables different dilution factors for saliva samples, and more importantly, has no interferences to thiocyanate and caffeine, which are quite abundant in saliva samples. It is cost-effective, requires equipment that is usually already available in laboratories and can be therefore performed by almost any laboratory.
